# Effect of prior pharmacotherapy on remission with sequential bilateral theta-burst versus standard bilateral repetitive transcranial magnetic stimulation in treatment-resistant late-life depression

**DOI:** 10.1192/bjp.2023.81

**Published:** 2023-11

**Authors:** Rafae A. Wathra, Benoit H. Mulsant, Zafiris J. Daskalakis, Jonathan Downar, Shawn M. McClintock, Sean M. Nestor, Tarek K. Rajji, Alisson P. Trevizol, Daniel M. Blumberger

**Affiliations:** Temerty Centre for Therapeutic Brain Intervention, Centre for Addiction and Mental Health, Toronto, Ontario, Canada; and Department of Psychiatry, Temerty Faculty of Medicine, University of Toronto, Toronto, Ontario, Canada; Department of Psychiatry, University of California, San Diego Health, San Diego, California, USA; Department of Psychiatry, University of Texas Southwestern Medical Center, Dallas, Texas, USA; Department of Psychiatry, Temerty Faculty of Medicine, University of Toronto, Toronto, Ontario, Canada; and Harquail Centre for Neuromodulation, Sunnybrook Health Sciences Center, Toronto, Ontario, Canada; Temerty Centre for Therapeutic Brain Intervention, Centre for Addiction and Mental Health, Toronto, Ontario, Canada; Department of Psychiatry, Temerty Faculty of Medicine, University of Toronto, Toronto, Ontario, Canada; Toronto Dementia Research Alliance, University of Toronto, Toronto, Ontario, Canada

**Keywords:** Depression, geriatric, rTMS, theta burst, neurostimulation.

## Abstract

Repetitive transcranial magnetic stimulation (rTMS) is used for treatment of late-life depression. In the FOUR-D study, sequential bilateral theta-burst stimulation (TBS) had comparable remission rates to standard bilateral rTMS. Data were analysed from the FOUR-D trial to compare remission rates between two types of rTMS based on the number and class of prior medication trials. The remission rate was higher in participants with ≤1 previous trial (43.9%) than in participants with 2 previous trials (26.5%) or ≥3 previous trials (24.6%; χ² = 6.36, d.f. = 2, *P* = 0.04). Utilising rTMS earlier in late-life depression may lead to better outcomes.

There is growing evidence supporting the use of repetitive transcranial magnetic stimulation (rTMS) in the treatment of late-life depression.^1^ Most rTMS protocols in late-life depression apply excitatory stimulation to the left dorsolateral prefrontal cortex (DLPFC) and some also deliver bilateral stimulation with an inhibitory stimulation to the right DLPFC prior to excitatory stimulation to the left DLPFC.^2^ Inhibitory stimulation has been delivered to the right DLPFC as this brain region has been shown to be overactive in people with depression.^2^ Newer rTMS protocols have used theta-burst stimulation (TBS), which can be delivered in less time and has been shown to be non-inferior to a conventional stimulation protocol involving high frequency (10 Hz) to the left DLPFC and low frequency (1 Hz) to the right DLPFC.^3^

Prior studies suggest that a greater number of unsuccessful medication trials predicts a poorer outcome of treatment, including electroconvulsive therapy^4,5^ and rTMS in younger adults.^6–8^ This suggests that patients with fewer previous treatments are more likely to respond to a new treatment. Treatment-resistant depression is prevalent in older adults,^9^ so it is important to determine the optimal period for using rTMS compared with other treatment approaches, such as augmentation pharmacotherapy.^10^

In the FOUR-D study, sequential bilateral TBS and sequential bilateral rTMS both had clinically meaningful remission rates (32.9% with conventional 10 Hz/1 Hz bilateral rTMS and 35.4% with TBS) in a large sample of adults with late-life depression.^11^ Using data from this trial, we sought to compare remission rates between bilateral TBS and bilateral conventional rTMS based on the number and class of medication trials in the current major depressive episode. We hypothesised that a larger number of prior trials would be associated with a lower remission rate with both rTMS modalities.

## Method

Data for this secondary analysis were from the FOUR-D study (ClinicalTrials.gov Identifier: NCT02998580), the details of which have been previously reported.^11^ In brief, it was a non-inferiority randomised controlled trial in out-patients, age ≥60, with a major depressive disorder and a current major depressive episode with a score ≥18 on the Montgomery–Åsberg Depression Rating Scale (MADRS), who had not responded to one adequate antidepressant trial or had been unable to tolerate two separate antidepressant trials. Participants were randomised to receive either standard right-sided DLPFC stimulation (120% resting motor threshold (RMT); 1 Hz frequency for 600 pulses over 10 min), followed by standard left-sided DLPFC stimulation (120% RMT; 10 Hz frequency; trains of 4 s on and 26 s off for 3000 pulses over 37.5 min) or continuous TBS (cTBS) of the right DLPFC (120% RMT; triplet burst pulses at 50 Hz; repeated at 5 Hz for 600 pulses over 40 s) followed by intermittent TBS (iTBS) of the left DLPFC (120% RMT; triplet burst pulses at 50 Hz; repeated at 5 Hz with a duty cycle of 2 s on and 8 s off; total of 600 pulses over 3 min and 9 s). All participants had rTMS daily for 20–30 weekdays. The authors assert that all procedures contributing to this work comply with the ethical standards of the relevant national and institutional committees on human experimentation and with the Helsinki Declaration of 1975, as revised in 2008. All procedures involving human participants were approved by the Centre for Addiction and Mental Health Research Ethics Board (approval number 076-2016). Written informed consent was obtained from all participants.

The number of adequate medication trials during the current depressive episode was determined using the Antidepressant Treatment History Form (ATHF).^9^ An adequate trial required the recommended therapeutic dosage of the medication for a duration of at least 4 weeks. Medication treatments given concurrently during rTMS were continuations of prior trials, as inclusion criteria included no changes in psychotropic medications in the 4 weeks preceding screening. For this analysis, remission was defined as a MADRS score ≤10. Pearson's chi-squared test was used to compare remission rates at the end-point of both treatment protocols between groups categorised based on treatment resistance. Fischer's exact test or Pearson's chi-squared test was used to compare remission rates in participants treated with specific classes of antidepressant. An intention-to-treat analysis was conducted, and those who dropped out of the study were assumed to have not achieved remission. All statistical analyses were conducted in IBM SPSS Statistics for Windows, version 26.

## Results

Of the 172 participants included in the analysis, 164 completed at least 18 treatments (Table 1). Prior to randomisation, 66/172 (38.4%) had one or no adequate medication trials, 49/172 (28.5%) had two adequate trials and 57/172 (33.1%) had three or more adequate trials. The remission rates were 29/66 (43.9%) in those with ≤1 adequate trial, 13/49 (26.5%) in those with 2 adequate trials and 14/57 (24.6%) in those with ≥3 adequate trials (χ² = 6.36, d.f. = 2, *P* = 0.04). The remission rates did not differ significantly for the different levels of treatment resistance in the sequential TBS (χ² = 4.61, d.f. = 2, *P* = 0.10) or standard sequential bilateral rTMS subgroups (χ² = 0.24, d.f. = 2, *P* = 0.24).

**Table 1:** Demographic and clinical characteristics and remission rates by number of previous adequate treatment trials

	Number of previous adequate treatment trials			
	≤1 (*n* = 66)^a^	2 (*n* = 49)	≥3 (*n* = 57)	Statistical test
Age, years: mean (s.d.)	66.7 (5.9)	66.8 (5.6)	66.5 (6.7)	
Self-reported female gender, *n* (%)	39 (59.1)	24 (49.0)	29 (50.9)	
Years of education, mean (s.d.)	15.5 (2.8)	14.5 (2.6)	15.7 (2.4)	
Previous ECT, *n* (%)	9 (13.6)	6 (12.2)	7 (12.3)	
Current psychotherapy, *n* (%)	23 (34.8)	15 (30.6)	20 (35.1)	
Age at onset, years: mean (s.d.)	45.4 (120.9)	29.4 (17.5)	32.4 (18.4)	
Episode duration, months: mean (s.d.)	54.1 (96.8)	62.2 (96.8)	67.1 (99.2)	
Antidepressant during treatment, *n* (%)	32 (48.5)	15 (30.6)	19 (33.3)	
Antidepressant combination during treatment, *n* (%)	7 (10.6)	8 (16.3)	18 (31.6)	
Augmentation during treatment, *n* (%)	6 (9.1)	17 (34.7)	17 (29.8)	
Lithium during treatment, *n* (%)	2 (3.0)	2 (4.1)	4 (7.0)	
Remitters in total sample, *n* (%)^b^	29 (43.9)	13 (26.5)	14 (24.6)	χ² = 6.36 (d.f. = 2, *P* = 0.04)
Remitters with TBS, *n* (%)^c^	15 (45.5)	8 (30.8)	5 (19.2)	χ² = 4.61 (d.f. = 2, *P* = 0.10)
Remitters with standard rTMS, *n* (%)^d^	14 (42.4)	5 (21.7)	9 (29.0)	χ² = 0.24 (d.f. = 2, *P* = 0.24)

ECT, electroconvulsive therapy; TBS, theta-burst stimulation; rTMS, repetitive transcranial magnetic stimulation.

a. 14/66 (21.2%) had no prior adequate antidepressant trial but were unable to tolerate at least two separate antidepressant trials.

b. In an analysis comparing remission rates for ≤1 *v*. >1 prior treatment trials, there was also a statistically significant difference: 29/66 (43.9%) *v*. 27/106 (25.5%) (χ² = 6.32, d.f. = 1, *P* = 0.02).

c. In an analysis comparing remission rates for ≤1 *v*. >1 prior treatment trials, there was no statistically significant difference: 15/33 (45.5%) *v*. 13/52 (25.0%) (χ² = 3.82, d.f. = 1, *P* = 0.051).

d. In an analysis comparing remission rates for ≤1 *v*. >1 prior treatment trials, there was no statistically significant difference: 14/33 (42.4%) *v*. 14/54 (25.9%) (χ² = 2.55, d.f. = 1, *P* = 0.156).

The most commonly used medications were selective serotonin reuptake inhibitors (96/172; 55.8%) and serotonin–noradrenaline reuptake inhibitors (89/172; 51.7%). A secondary analysis revealed that the remission rates did not differ significantly in the groups previously treated with a specific antidepressant class.

## Discussion

In this analysis, the remission rate was higher in participants treated with bilateral rTMS who had not responded to ≤1 medication trial than in those who had not responded to ≥2 trials. The remission rates did not differ in the study arms treated with bilateral TBS or conventional bilateral rTMS based on the level of treatment resistance or in the subgroups based on the class of prior medications.

Our results are consistent with previous studies reporting that a lower level of treatment resistance is associated with better rTMS treatment outcomes.^2^ Also, our finding of no difference in remission rates with bilateral TBS or conventional bilateral rTMS based on the number of prior treatment trials confirms that both forms of rTMS have similar clinical efficacy.^7^ Of note, there were no differences in remission rates between participants who had not responded to two or more than two prior medication trials. This suggests that the optimal timing to consider rTMS treatment may be after the first failed medication trial, to maximise chances of remission.

A limitation of this study is that the number of prior medication trials is possibly confounded by the duration of the depressive episode. Future studies should try to disambiguate the relationship between episode duration and prior medication trials. In addition, participants with a greater number of prior medication trials had a higher prevalence of concurrent antidepressant combinations and augmentation pharmacotherapy during treatment. This likely reflects the treatment strategies used in treatment-resistant depression.

Our findings support that a detailed assessment of treatment history is an essential component of assessment for rTMS suitability. Our results also suggest that rTMS should be considered earlier in the treatment of depression in older adults to potentially reduce the likelihood of developing treatment-resistant depression. Until personalised medicine is fully incorporated into psychiatry it is important to use treatments with different mechanisms of action earlier in the care of older adults with depression. As data would suggest that such approaches are more effective, rTMS could potentially be incorporated earlier in the treatment algorithm to maximise its potential for efficacy. Furthermore, our findings further support the non-inferiority of bilateral TBS to conventional bilateral TMS in late-life depression, as remission rates based on treatment resistance did not differ in the two study arms. In the future, a composite score of clinical information and biomarkers might be used to identify patients who are more likely to benefit from rTMS earlier in their treatment.

## Data Availability

The data that support the findings of this study are available from the corresponding author, D.M.B., on reasonable request.
